# New neurons flatten social hierarchies

**DOI:** 10.1038/s41598-026-57994-1

**Published:** 2026-06-23

**Authors:** Birte Doludda, Marcel Franz, Jadna Bogado Lopes, Rupert W. Overall, Annette E. Rünker, Ilona Croy, Gerd Kempermann

**Affiliations:** 1 CRTD - Center for Regenerative Therapies, TU Dresden, Dresden, Germany; 2 German Center for Neurodegenerative Diseases (DZNE) Dresden, Dresden, Germany; 3 Department of Clinical Psychology, Institute of Psychology, Friedrich- Schiller-University Jena, Jena, Germany; 4 Institute of Biology, Humboldt University, Berlin, Germany; 5 German Centre for Mental Health (DZPG), Halle-Jena-Magdeburg, Germany; 6 Department of Psychotherapy and Psychosomatic Medicine, Faculty of Medicine, University Hospital Carl Gustav Carus, TU Dresden, Dresden, Germany

**Keywords:** Neuroscience, Psychology, Psychology

## Abstract

As complex individualized behaviors in mice arise even when genes and environment are held constant, emerging social group structures should also depend on individual behavior. Adult hippocampal neurogenesis drives individual behavior and is involved in certain social behaviors. Would thus the emergence of social structure depend on adult-born neurons? To answer this question, we co-housed cohorts of female mice with intact and genetically diminished general adult neurogenesis in a large-scale automated tracking system for three months and analyzed the development of social behaviors over time. Not only did female mice develop stable hierarchies, we also found that, despite forming a functioning mouse society, their dominance behavior increasingly diverged over time. Specifically, neurogenesis-deficient mice showcased a greater increase in hierarchical behavior throughout the experiment. Within the social behaviors, mice showed no genotype-based preference, indicating intrinsic causes for behavioral differences. In mice with intact neurogenesis, social rank was positively correlated with adult hippocampal neurogenesis. Nevertheless, neurogenesis-deficient mice occupied higher ranks in the emerging hierarchy than their wild-type cohabitants, while displaying less individualization for this trait. The observed insistence on dominance might therefore reflect inflexible social strategies. Thus, social intelligence and flexibility, enabled by adult-born neurons, promote social adaptability, egality, and flatter hierarchies.

**Supplementary Information:**

The online version contains supplementary material available at 10.1038/s41598-026-57994-1.

## Introduction

Effective beneficial social behaviors require cognitive flexibility, as sociality depends on constant adaptive processes between individuals. The hippocampus, as a main integrative center in learning and memory, is critical for social behaviors^1^ and adult-born neurons in the hippocampus contribute to social behaviors^2,3,4^. But does this hold true for ethologically relevant complex behavior in large groups and over time?

In enriched environments (ENR), mice are exposed to sensory, motor, social and cognitive stimuli, which results in increases in adult hippocampal neurogenesis (AHN) and brain connectivity, and changes behavior^5^. ENR offer more opportunities for social contacts and lead to more complex and diverse social networks^6,7^. However, not all individuals realize the given opportunities in the same way, which generates heterogeneity in behavior, which in turn should result in social patterns.

The hippocampus is involved in flexible cognition and social behavior^1^, and emerging hypotheses emphasize a causal link between the two aspects. In fact, cognitive flexibility facilitates and supports social behavior. Specifically, the CA2 region of the hippocampus, which plays an important role in social memory^8,9^, and AHN occurring in the dentate gyrus, a key example of plasticity that supports cognitive flexibility^10,11,12^, have both been linked to several aspects of social behavior^13,14^. AHN provides cognitive plasticity and flexibility in terms of behavioral pattern separation, the update of cognitive maps, and the contextualization of information^15^, all of which are important for social learning^2,3^. However, when animals experience social isolation, these mechanisms can be disrupted, as isolation has been shown to reduce AHN^16,17^. In addition, AHN has been linked to the mediation of the effects of stressors such as social isolation^18^.

**Fig. 1:**
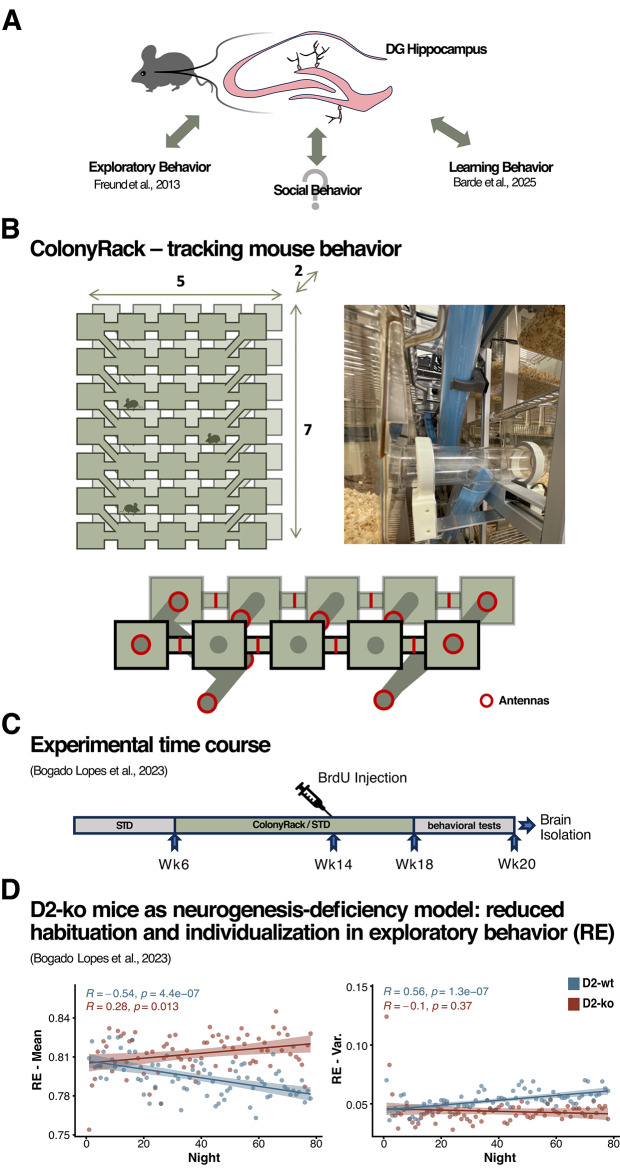
(**A**) Concept of the influence of ENR components on AHN. (**B**) A schematic of the ColonyRack, composed of 70 interconnected standard mouse cages with a total of *n* = 115 antennas at connecting tubes that enable tracking of large groups of mice with embedded RFIDs. (**C**) The time course of the experiment. (**D**) The mean (left) and standard deviation (var.; right) of the RE exploratory behavior developing over 78 nights (revised data) in D2-wt (blue, *n* = 44) and adult neurogenesis-deficient D2-ko mice (red, *n* = 37) analyzed by Bogado Lopes et al. 2023^29^. Linear regression p and r values are reported.

Our previous research suggests a likely causal link between AHN and two behavioral dimensions: the individualization of exploratory behavior, primarily reflecting motor aspects, and of learning behavior, representing cognitive components (Fig. 1A;^19,20,21^. We here turn to the question, to which extent social behavior similarly shows signs of increasing individualization, even when genetics and shared environment are controlled and to which extent this pattern is dependent on adult neurogenesis. Using cyclin D2 knock-out mice, in which adult neurogenesis is impaired in both the hippocampus and the subventricular zone ^22,23^, we may not be able to attribute social abnormalities to neurogenesis in specifically either of these regions. However, several studies investigating the relationship between male rodent social hierarchies and AHN have reported higher levels of AHN in more dominant individuals^4,24^, which led us to hypothesize that AHN is indeed involved in hierarchical behavior and thus should contribute to social behaviors measurable in our setting, even though other neurogenic and non-neurogenic factors might contribute as well.

To date, essentially all studies on social structure in laboratory rodents, have suffered from low temporal resolution, small group size, short duration, and the inference of social structure from pair-wise confrontation or other experimenter-dependent tests. Under those conditions, however, the questions could not be addressed, how complex social structure at large would develop over a period of time and, consequently, how individual neurogenesis-dependent behavior would affect social structure such large group of mice.

Generally, murine studies on social activity have used behavioral tests, ethograms, and video data with machine learning^25,26,27^, which cannot be applied to longitudinal settings in larger cohorts of mice. The use of behavioral tests, such as single tube dominance tests or the warm spot competition task require removing the animals from their normal cage setting into a novel environment and situation, potentially obscuring or disturbing natural patterns of behavior and hierarchy. In contrast, using an automated home-cage tracking system consisting of 70 interconnected standard cages equipped with radio-frequency identification (RFID) antennas (Fig. 1B), we can track large groups of – usually female, due to their low territoriality – mice over months with a spatial resolution of the individual cage and a temporal resolution of less than one second, enabling us to monitor behavioral patterns in space and their dynamics^20,28^. From the resulting datasets we can perform continuous analyses on the development of social behaviors and later also relate these to adult neurogenesis. As social structures underly all populational activities, whether in rodents or humans, this methodology can promote our understanding of the emergence of social dynamics and the plasticity of traits present in brain and behavior, while controlling genetic effects (through the use of inbred mice) and the nominal external environment (“shared environment”). Specifically, our aims were to decipher (1) to which extent information about social behavior and structure could be derived from the temporo-spatially coded activity data from our cage system, (2) which types of hierarchy form, (3) how social patterns change over time, and (4) how social behavior is impacted by constitutive lack of adult neurogenesis.

## Results

We previously^29^ co-housed female cyclin D2 (D2) wild-type (D2-wt, *N* = 44) and D2 knock-out mice (D2-ko, *N* = 37) (Sicinski et al. 1996)^30^ – an established, well characterized model with constitutively minimal adult neurogenesis in the hippocampus Kowalczyk^23,31,32^ and the subventricular zone^22^ – for 85 days in our cage system (Fig. 1C). Exploratory behavior expressed as roaming entropy (RE) increasingly differed between D2-ko and D2-wt mice (Fig. 1D; data reanalyzed from our previous study, Bogado Lopes et al.^29^). In D2-ko mice, individualization of RE trajectories was attenuated, as indicated by the lack of increase in variance that was observed in D2-wt (r(76) = − 0.1, *p* = 0.37; data reanalyzed from our previous study Bogado Lopes et al.^29^; Fig. 1D), consistent with the findings from mice with normal adult neurogenesis reported in other studies^28^.

**Fig. 2:**
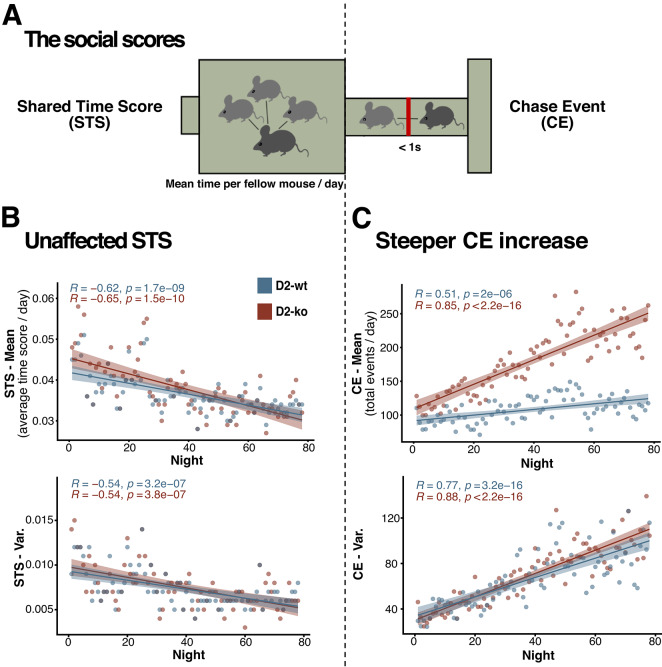
**(A)** A schematic of the social scores analyzed for the metrics, SCT and CE. **(B)** The mean and SD of the shared time score (SCS) behavior developing over 78 nights in D2-wt (blue, *n* = 44) and D2-ko mice (red, *n* = 37) **(C)** The mean and SD of the chase events (CE) behavior developing over 78 days in D2-wt (blue, *n* = 44) and D2-ko mice (red, *n* = 37). Linear regression p and r values are reported.

From the tracking data, also rich information about social interaction can be derived. As sociality is composed of individual, yet interacting trajectories, we first calculated shared time score (STS) and chase events (CE) as indicators of social activity (Fig. 2A). STS is a normalized value for the average daily time a target mouse spends in the same cage with each of its fellow mice. CE are scored for a target mouse that triggers the same antenna within one second after another mouse. These events are recorded for each mouse per day (Suppl. Figure 1A for the association between different metrics; including the amount of time spent alone).

**Fig. 3:**
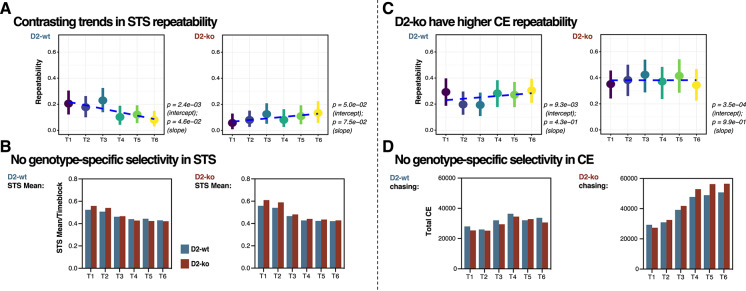
**A**. The repeatability calculated for the mean STS in D2-wt (44, left) and D2-ko (37, right). Significance of intercept and slope were calculated from linear mixed model with 95% confidence interval. **(B)** The average STS of D2-wt (44) or D2-ko (37) spent with either D2-wt or D2-ko in different time blocks (*n* = 13 days). **(C)** The repeatability calculated for the mean CE in D2-wt (44, left) and D2-ko (37, right). Significance of intercept and slope were calculated from linear mixed model with 95% confidence interval **(D)** The total CE of D2-wt (44) or D2-ko (37) directed either toward D2-wt or D2-ko in different time blocks (*n* = 13 days).

In both genotypes, we found a comparable decrease in the mean and variance (or SD) over time for STS (Fig. 2B), consistent with the idea of social habituation. Additionally, there was no genotype-dependent specificity in STS: both genotypes spent time with each other (Fig. 3B). Together these findings indicate a functioning murine society. The lack of a difference between genotypes and of a correlation of STS with AHN in D2-wt mice (Suppl. Figure 2 C) argues against an involvement of AHN in this parameter of social activity.

In contrast, the increase in CE over time in both genotypes, was significantly more pronounced in D2-ko compared to D2-wt (Fisher’s z, z = -4.35, *p* < 0.0001; Fig. 2C), while the variance of the CE trajectories was similar (Fig. 2C).

Looking at the repeatability of their behaviors, i.e. how consistent the mice in a group are in their behavior over time above intraindividual fluctuations^21,28^, both D2-wt and D2-ko mice showed low repeatability in STS behavior (Fig. 3A). However, whereas repeatability in D2-wt mice STS behavior continued to drop throughout the experiment, it increases slightly in D2-ko mice. With regard to their CE behavior, D2-ko mice showed a higher repeatability compared to D2-wt mice (Fig. 3C). However, while repeatability increased for D2-wt mice, there was no significant change in D2-ko mice, which remained high throughout the experimental period.

**Fig. 4:**
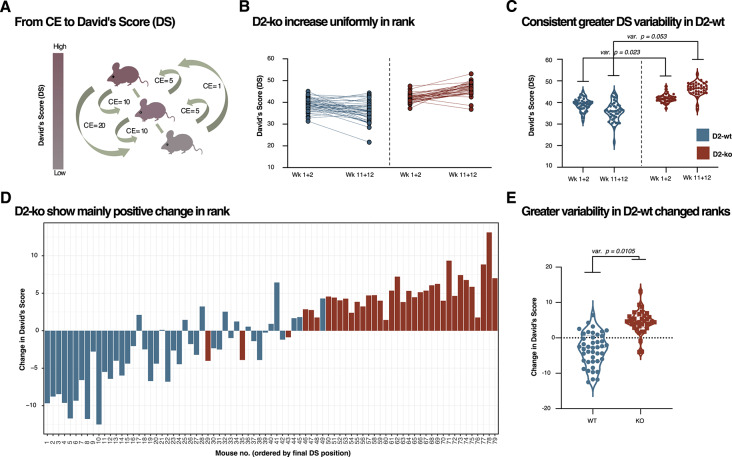
**A.** an overview of going from CE to the David’s score (DS). **B** The DS calculated for the first two weeks (*n* = 13) and the last two weeks (*n* = 14) of the experiment for D2-wt (blue, *n* = 43) and D2-ko (red, *n* = 36). **C.** The violin plot shows the variance in the first two weeks DS and last two weeks DS. Variance was compared with Levene’s test, p values reported. **D.** The change in DS calculated by subtracting the DS for first two weeks (*n* = 13) from the DS from last two weeks (*n* = 14) of the experiment for D2-wt (blue, *n* = 43) and D2-ko (red, *n* = 36). **E.** The violin plot shows the variance in the change in DS. Variance was compared with Levene’s test, p values reported.

Next, from the CE score, dyadic agonistic interactions were extracted enabling us to calculate social hierarchies^33^. There was no specificity in the directionality of chasing: both genotypes chased and were chased (Fig. 3D). From the total number of encounters the David’s score (DS)^34^ for each mouse was calculated. The DS is an established metric for determining dominance ranks, which is usually derived from few deliberately arranged pair-wise encounters, but can be drawn here from the average number of spontaneous confrontations between any two mice (Fig. 4A). This improvement in resolution and reliability allowed us to study hierarchy patterns and their more subtle modulation over time.

Comparing the DSs from the first with the last two weeks of the experiment revealed differences in the hierarchy patterns and dynamics between genotypes. D2-wt mice had statistically significantly lower hierarchy ranks (DSs) than D2-ko mice at the start (Wilcoxon test, D2-ko: *n* = 36, *Mdn* = 41.7, D2-wt: *n* = 43, *Mdn* = 39.5; W = 1240, *p* = 1.827e−06, *r* = 0.516; Fig. 4B) and at the end of the experiment (Wilcoxon test, D2-ko: *n* = 36, *Mdn* = 46.6, D2-wt: *n* = 43, *Mdn* = 36.1; W = 1517, *p* < 2.2e−16, *r* = 0.823; Fig. 4B). However, the variance of DS positions was higher in D2-wt compared to D2-ko mice, again both at the start (Levene’s test, F(1,77) = 5.35, *p* = 0.023) and the end (Levene’s test, F(1,77) = 3.88, *p* = 0.053; Fig. 4C). Taken together, this means that D2-wt mice maintained a broader range of DS positions at lower levels, while D2-ko mice aspired a narrower range of high hierarchy positions. Such a cohort-wide dominance phenotype in female mice resulting from a single-gene knockout (and thereby the lack of AHN and diminished SVZ-based adult neurogenesis) has not yet been described previously.

Looking further at the relationship between the early and late DS ranks, the early DS ranks showed a stronger Spearman correlation coefficient in D2-ko than in D2-wt mice (Suppl. Figure 3). While the D2-ko showed a strong correlation early, this link appeared only later in the wildtype mice. This might indicate that the D2-wt adjusted to the social situation over a longer period of time. Together, this suggests that the D2-ko mice settled on their ranks quicker than the D2-wt mice, leaving the latter to adjust their ranks based on the other mice in their environment.

We next analyzed the individual gains and losses in hierarchy rank between the early and late test periods (change in DS; Fig. 4B), which revealed a segregation according to genotype (Fig. 4B). While the majority of D2-ko mice (91.7%) increased their DS and established themselves at the top of the hierarchy, only a minority of D2-wt mice moderately improved their rank (27.9%) and most substantially lost in rank position. With this, the mean change in DS rank was significantly lower (Wilcoxon test, D2-ko: *n* = 36, *Mdn* = 4.51, D2-wt: *n* = 43, *Mdn* = -2.64; W = 1428, *p* = 7.715e−13, *r* = 0.724) but also more variable for D2-wt mice compared to D2-ko mice (Levene’s test, F(1,77) = 6.8814, *p* = 0.0105; Fig. 4E).

In summary, AHN, absent in D2-ko mice, might provide the cognitive flexibility required to adapt to the – in this case persistently dominant – social environment and allow for greater variability in individual strategies in determining rank. On the other side, diminished cognitive flexibility in D2-ko mice might impair their ability to cope with the complex social network of a large group of mice and to adjust their behavior, resulting in more stereotypical aggressive behavior^35^.

The dominant behavior of D2-ko mice might theoretically also arise from attempts to compensate for increased anxiety. However, an open field test (OF) revealed similar time spent in the center (OF1: Wilcoxon test, D2-ko: *n* = 38, *Mdn* = 43.7, D2-wt: *n* = 44, *Mdn* = 39.4; W = 922, *p* = 0.4286, *r* = 0.0883; OF2: Wilcoxon test, D2-ko: *n* = 38, *Mdn* = 13.6, D2-wt: *n* = 44, *Mdn* = 10.5; W = 1017.5, *p* = 0.09235, *r* = 0.186; Suppl. Figure 2D) indicating no differences in anxiety between the genotypes, at least with this coarse measure. This finding in female mice is in contrast to reports suggesting effects of hierarchy on anxiety levels in males^36,37,38^.

**Fig. 5:**
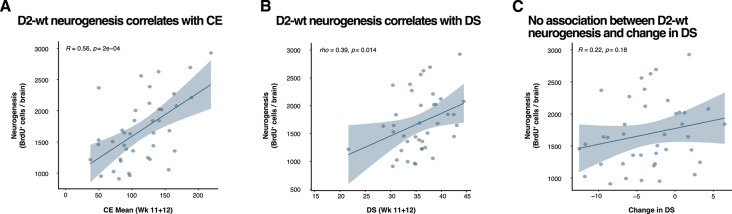
Pearson correlations with number of BrdU positive cells with CE mean of the last two weeks and change in DS, and spearman correlation of DS (wk 11 + 12) and number of BrdU positive cells for D2-wt (blue, *n* = 40). Respectively, r and p values and rho and p value is reported.

As deficiency in adult neurogenesis thus apparently had a profound effect on dominance behavior, we further investigated the relationship between plasticity and behavior in D2-wt mice. We labeled new-born neurons in the hippocampal dentate gyrus with bromodeoxyuridine (BrdU; 4 weeks survival after injection) and found significant positive correlations between BrdU-positive cell counts and the number of CE as well as the DS position (Fig. 5A, B and r(38) = 0.56, *p* = 2e−04; r(38) = 0.39, *p* = 0.014), while the correlation between new neurons and the change in DS was not significant (Fig. 5C). To strengthen this claim, bootstrap analyses for CE and BrdU-positive cells, with 2000 resamples, yielded a 95% bias-corrected and accelerated (BCa) confidence interval of 0.2180–0.7532, indicating a reliable positive association. For DS and BrdU-positive cells, 2000 resamples yielded a 95% BCa confidence interval of 0.1063–0.5583.

Such a connection between hierarchy position and AHN is in line with the predictions from studies based on short term-paradigms in small groups^4^.

## Discussion

We here report that (1) rich information about social behaviors and structure and their dynamics can be extracted from longitudinal tracking data, (2) female mice in larger groups develop stable hierarchies, which (3) adjust over time, and (4) depend on brain plasticity. Hierarchy position and AHN were positively correlated in D2-wt littermates.

However, impaired brain plasticity appears to result in more rigid behavioral strategies that involve an ongoing effort to (aggressively) defend the rank. This is evident from a stronger final-rank correlation formed in the early time block in mice with impaired adult neurogenesis, while the mice with intact plasticity showed lower strength, indicating greater rank shifts during this time. Mice lacking adult neurogenesis thus paradoxically occupied the highest positions in the hierarchy and dominated over the rest of the mice, while AHN, when present, provided the flexibility to adapt, including adaptation to the more aggressive behavior. Adult neurogenesis and cognitive flexibility thus allowed the mice to flexibly adjust their rank, albeit at a more submissive level, yet thereby forming a flatter and more permeable hierarchy within their group.

These results are consistent with a study suggesting that suppressed AHN in adolescent male mice increased dominance and anxiety-related behaviors as measured by conventional behavioral tests^24^. Notably, since female mice were used here, there is a similarity in phenotypes between the sexes, even though it has been reported that females display less hierarchical behavior^39,40^. There are additional reports that the type of hierarchy differs between sexes, with males having more despotic hierarchies, while female hierarchies being less linear and steep^39^. Repeating our experiment in males would not only further validate those findings, but also allow the analyses of social structure development in the more aggressive sex.

D2-ko mice have a reduced ability to form long-term memories most likely due to the lack of adult neurogenesis in the hippocampus, while short term memory is intact^31^. Specifically, new neurons are here involved (among other functions) in facilitating the integration of new information into pre-existing contexts and thus cognitive flexibility^10,12^. In line with this, new-born neurons have been suggested to be required for social memory maintenance^41^. The hyperdominant behavior of D2-ko females in our study might therefore be partly the result of reduced social memory, required to remember individuals in an established hierarchy. The impaired ability to cope with novel situations^42^ might contribute to the stereotypical preference for dominant behavior in D2-ko females^14^.

There is evidence for a reduced size of the olfactory bulb and/or diminished subventricular zone-based adult neurogenesis in D2-ko mice, hindering their ability to find food hidden deeper in the bedding^43^. We found evidence of remaining proliferation within the olfactory bulb of D2-ko mice, in contrast to the almost complete loss within the hippocampus, supporting the idea of olfaction being impaired but not completely lost (Suppl. Figure 4). As olfaction plays a role within social context, the degree to which an impairment in olfaction affects their social behavior should be considered in the interpretation of these results.

In D2-wt, the number of new neurons in the hippocampus reflected hierarchy position, with more hippocampal neurogenesis at higher ranks (Fig. 5B). Nevertheless, adult neurogenesis differentially affected various social aspects, as STS was similar between genotypes, which appears to be based on social skills other than those important for attaining and maintaining rank. These skills in D2-wt presumably involve more cognitive (AHN-dependent) mechanisms, whereas in D2-ko they are more reactive and aggression-based. A study in which wild-derived outbred or C57BL/6J mice were housed in sex mixed groups in an outdoor field enclosure with behavioral tracking, reported higher social activity in C57BL/6J females^44^. However, stronger interactions among males were related to hierarchical behavior. Vogt et al. found that males of both mouse strains showed territorial behavior rather than behavior leading to an alpha-dominated hierarchy, although this behavior was muted in C57BL/6J male mice. The exact nature of the hierarchical interactions and the extent to which these were territory-related is unknown for their experimental system. In addition, dominance behavior of females was not assessed. However, our data on female mice indicate an inherent drive to generate a hierarchy that goes beyond territorial behavior and leads to the formation of a functioning society. Whether this is due to the limited space of our cage system compared to the open field setting used by Vogt et al., remains an open question. Territoriality seemingly also plays a role in female dominance behavior^45,46^. Male mice appear to identify ranks of known and unknown other mice using a combination of olfaction and chemosensation^47^. It is likely that in female mouse colonies, the method of rank sensing is similar. This would intuitively suggest that better rank recognition or social memory allows for a flexible adjustment of individual dominance behavior towards different social mates, which requires further investigation. In fact, AHN has been shown to be essential for social memory^41^. Curiously, we did not observe any selectivity in shared time spent with one of the two genotypes in mice with intact adult neurogenesis. Even with intact social memory, increased aggressive and hierarchical behavior did not cause the mice to develop a preference for the less hierarchical mice; interactions continued to be non-selective, which is in line with the findings in Vogt et al. of lower selectivity for social partners in C57BL/6J female mice despite a denser social network. Thus, the increased desire for sociality might overpower the aversive affect caused by increased hierarchical chasing behavior^44^.

The described association between AHN and social structure does not imply direct causation in either direction: Whether increased neurogenesis facilitates higher rank attainment, or higher rank attainment facilitates higher amounts of neurogenesis remains a question. This likely comes down to a reciprocal relationship, integrating spatio-temporal circumstances created by intrinsic and extrinsic factors. Our study goes beyond previous work by demonstrating a link between hierarchy and neurogenesis in female mice, complemented by the counter-intuitive observation that lacking adult neurogenesis results in stereotypical, inflexible hierarchical behavior that secures the top rather than the bottom hierarchical ranks.

As highlighted in Fig. 1A, multiple behavioral patterns have already been connected to AHN, such as exploration and learning^19,21^. This research provides evidence for the further connection of a social behavior to adult neurogenesis. The added dissection of social behavior into shared time and chasing, highlight the specificity or importance in one social aspect over another as we did not find any connection between the shared time and AHN. Both chasing and exploration contain a physical activity aspect, making the normalization of chasing by exploration (Suppl. Figure 2 C) vital as it shows that even with a consideration of exploration, chasing significantly correlates with AHN. Thus, different facets create a combined effect, vital for neurogenesis. Further dissecting which aspects connect to neurogenesis, provide us with valuable insight to transfer these findings to humans.

It is important to note that the model used in this experiment, is not selective for ablating AHN, but also affects other neurogenic niches, such as the subventricular zone. Developmental neurogenesis in the D2 mutant mice results in the formation of the major cerebral structures with grossly normal morphology, albeit with a reduction in size^23,31,48,49^. Nevertheless, D2-ko mice behave normal in terms of motor function and coordination, sensorimotor skills, anxiety level, procedural learning, hippocampus-dependent spatial learning, and, as highlighted within our experiment, a number of aspects of social behaviors^31,32,43,50^. This, in combination with other similar findings^4,24,51,52^, suggests that AHN might be particularly important for hierarchy-related social behavior, even though we cannot exclude the contribution of other factors. Unfortunately, there is no model available to selectively ablate adult neurogenesis in the hippocampus, which is compatible with our hands-off long-term experiments.

The key advantage of our automated cage set-up is to allow tracking of individuals in a large cohort of mice and the development of their social structures over prolonged periods of time. The ability to dissect behavior longitudinally with a daily resolution allowed the determination of a time frame in which there was a greater stability in the established ranks in one group compared to another. Such differentiation would have been close to impossible using traditional hierarchical ranking methods, such as the single-tube dominance test. Covering extended periods of time in a large cohort of mice would have required the daily extraction of the animals from the system, cross individual tests taking greater hours and manual work-load, and introduced a great amount of external stress to the animals. Thus, the automated home-cage presents a system that specifically allows for longitudinal social analyses with the resolution of individual animals and the assessment of dynamic change.

Our findings suggest a fundamental neurobiological mechanism underlying social behavior and brain plasticity on one hand, and the relationship between social and cognitive rigidity on the other^53^.

## Methods

### Animals and housing conditions

Animals and housing conditions are described in Bogado Lopes et al. (see for more details)^29^. We confirm that all animal experiments were done in accordance with the ARRIVE guidelines and conform to the applicable European and national regulations (Tierschutzgesetz) and were approved by the local authority (Landesdirektion Sachsen; TVV 58/2016, File DD24-5131/354/63). In brief, the mice were attained from the Nencki Institute, Poland and were housed at the CRTD, Dresden. The mice were randomly distributed at 5 weeks of age in STD (not used here) and ENR categories. ENR animals (44 D2-wt and 40 D2-ko) were then injected with RFID tags and co-housed together in the ColonyRack (Phenosy GmbH). 44 D2-wt and 37 D2-ko were then used for analyses, due to some D2-ko animals showing incomplete behavioral data. Every two weeks, the animals were removed, the cages were cleaned, and toys were changed. Four weeks before the end of the experiment, the mice were injected with BrdU (50 mg/kg, Sigma) to label newborn neurons. Mice were euthanized with a mixture of ketamine as anesthetic agent (100 mg/kg) and xylazine (10 mg/kg for sedation, anesthesia and muscle relaxation) and then transcardially perfused with 0.9% saline and 4% paraformaldehyde (PFA) in phosphate buffer (pH 7.4).

### Tissue preparation and immunohistochemistry

Tissue preparation and immunohistochemistry are described in Bogado Lopes et al. 2023 (see for more details)^29^. In brief, brains that were stored in PFA (4%) were placed in sucrose (30%) before being sectioned using a Leica microtome into 40 μm sections. These were then stained for BrdU using the peroxidase method. Free-floating sections were incubated in hydrogen peroxide (0.6%) for 30 min, followed by incubation in hydrochloric acid (2.5 M) for 30 min at 37 °C. Then sections were incubated for 1 h at room temperature in tris-buffered saline (TBS) supplemented with Donkey Serum (10%; Jackson Labs) and TritonX-100 (0.2%; Carl Roth). Then, sections were incubated with primary antibody (monoclonal rat anti-BrdU 1:8000; ab6326, Abcam) overnight at 4 °C, washed, and then incubated with secondary antibody (Jackson ImmunoResearch Labs) for 2 h at room temperature. Antibody was always diluted in TBS with Donkey Serum (3%) and TritonX-100 (0.2%). Detection was then done using Vectastain Elite ABC Reagent (9 µL/reagent; Vector Laboratories, Linaris) and diaminobenzidine (0.075 mg/mL; Sigma-Aldrich) and nickel chloride (0.04%). Washing was done with TBS. Stained sections were then mounted on slides, cleared with Neo-Clear (Millipore) and cover-slipped with Neo-Mount (Millipore). Counting of BrdU positive cells was done using every 6th section along the rostro-caudal axis of the dentate gyrus and finally multiplied by 6 to have the total cell number/dentate gyrus of the hippocampus.

### Open field test

For the Open Field Test (as part of the Novel Object recognition Test Protocol), animals were placed in a white polycarbonate plastic arena (30 cm × 30 cm × 40 cm) and allowed to explore the open field for a period of 5 min at a time. Afterwards, the animals were removed and placed in their home cage.

### Analyses and statistics

For the analysis of the behavioral data, RFID readings were saved in csv files and processed using the ColonyTrack package (by Rupert Overall, 1.0.5 https://rupertoverall.net/ColonyTrack/index.html) in Rstudio. (Team R, 2022). Doing so, only night data was extracted, and cleaning days were removed. The data was then further processed to isolate the cage time entropy (RE), follow events (chase events, CE), social interaction (shared time score, STS), and time alone details. RE is the amount of exploratory activity calculated using the formula:

$$\:\frac{-{\sum\:}_{i=1}^{n}{p}_{i}\cdot\:\mathrm{log}\left({p}_{i}\right)}{\mathrm{log}\left(\mathrm{n}\right)}$$

With p being the fraction of the time spent in each compartment and n the number of compartments.

CE is defined as one mouse triggering an antenna within 1 s of another mouse having triggered the same antenna, going into the same direction. STS is calculated using the amount of time one mouse spends in the same cage compartment as all other mice—a normalized mean value is reported. For the purpose of looking at group averages and variance, the mean and SD was calculated separately for each group for each day. For repeated measures (longitudinal data) of the RE, CE and STS, a linear regression was performed using the lm function from the ‘stats’ package, and R and p values were obtained and listed. The David score (DS) was calculated based on CE and normalized using the package ‘steepness’ in Rstudio.

For the purpose of this, chasing events of the weeks were summed and the normalized DS calculated for the beginning (weeks 1 and 2) and end (weeks 11 and 12). Change in DS was calculated by taking the difference between the last time points and first time points. For correlations, first tests of normality were performed using Shapiro wilk test. Based on these results, either Pearson (normal) or Spearman (non-normal) correlations were performed. For variance calculations Levene’s test was used. Wilcoxon rank sum test was used to compare means. For comparison of correlations, Fisher’s z test was used. Genotype specificity was determined by looking at each animal’s interactions with each other animal (separated by genotype) separately and then summed or averaged. For the purpose of the DS analyses, two outliers, as determined by Grubbs’s test, were excluded from further analyses. Visualizations were made using Prism 9 (Graphpad) and ‘ggplot2’ in Rstudio. Repeatability was calculated using the package ‘rptR’ in Rstudio. In brief, repeatability considers the inter- and intra-variability proportions, by dividing inter- variability by the sum of inter- and intra- variability. Thus it shows the closer a score goes to 1, the less intra-variability is responsible for the variability seen in behavior and so the mice greater stable behavioral repeatability on an individual level. For strength in claim between BrdU positive cells and certain behavioral scores, bootstrapping was performed using the R package ‘*boot’*. The 95% bias-corrected and accelerated (BCa) confidence interval are given.

## Supplementary Information

Below is the link to the electronic supplementary material.

Supplementary Material 1

## Data Availability

The behavioral data are assessable here (CC BY-NC 4.0): [https://doi.org/10.25532/OPARA-1024](https:/doi.org/10.25532/OPARA-1024).
