# Supplementary material for: New neurons flatten social hierarchies
Source: Sci Rep. 2026 Jun 23;16:19541. doi: 10.1038/s41598-026-57994-1 (PMC13291321; doi:10.1038/s41598-026-57994-1)
Supplement: Supplementary file 1 — Supplementary Material 1 [file 41598_2026_57994_MOESM1_ESM.pdf]

Suppl. Material

Doludda et al. “New neurons flatten social hierarchies”

Suppl. Fig 1

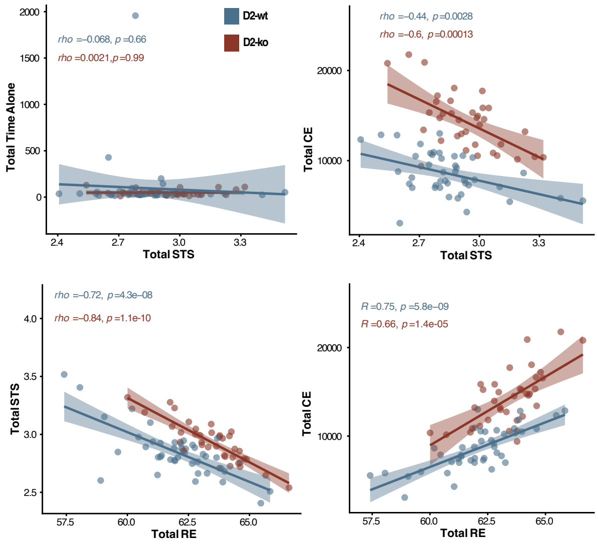

Suppl. Fig .1: Top left: Spearman correlation between the total time alone and the total STS. Top right: Spearman correlation between the total CE and the total STS. Bottom left: Spearman correlation between the total STS and the total RE. Bottom right: Pearson correlation between the total CE and the total RE. In all graphs, values of individual D2-wt (n=43) and D-ko (n=37) mice for 78 nights are shown as dots and respectively Spearman or Pearson correlation values are reported.

Suppl. Fig 2

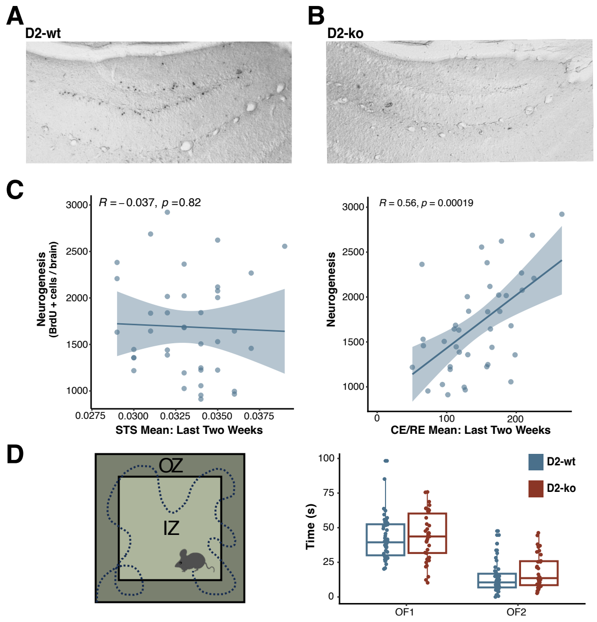

Suppl. Fig. 2: **A./B.** Representative images of BrdU-positive cells in the dentate gyrus of D2-wt (**A**) and D2-ko mice (**B**). **C.** Left: Pearson correlation between STS average of the last two weeks (n=14) and the BrdU-positive cell count for D2-wt mice (n=40). Right: Pearson correlation between the ratio of CE to RE means from the last two weeks (CE/RE mean; n=14) and the BrdU-positive cell count in D2-wt mice (n=40). **D.** Time spent in the open field or inner zone (IZ) in the open field test of D2-wt (n=42) and D2-ko (n=37) mice in trial 1 (OF1) and trial 2 (OF2).

Suppl. Fig 3

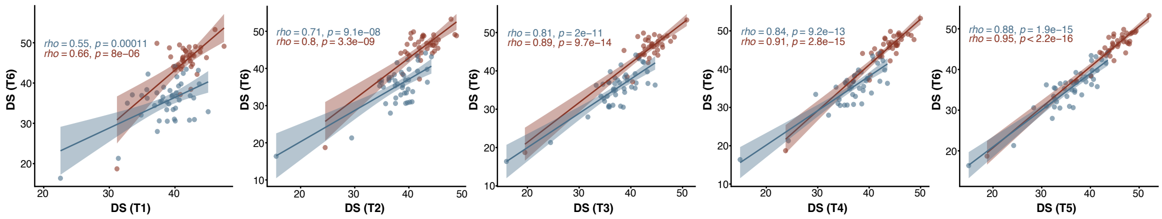

Suppl. Fig. 3: Spearman correlations for D2-wt (n=44, blue) and D2-ko (n=37, red) mice between their DS rank in the final time block (T6, n=13) and the DS rank in each other time block (5 time blocks T1-T5, n=13 each). Rho and p value are reported.

Suppl. Fig 4

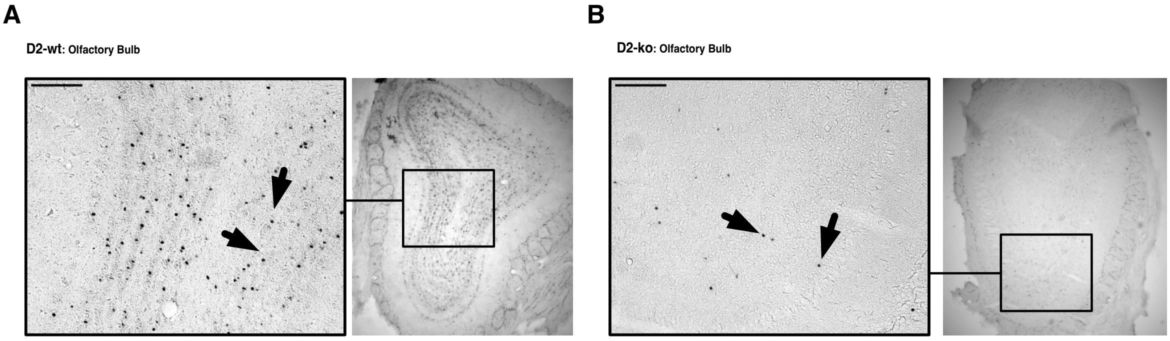

Suppl. Fig. 4: Representative images of BrdU-positive cells in the olfactory bulb of D2-wt (**A**) and D2-ko (**B**) female mice. Scale bar 100um.
